# Recombinant Human Thyroid Stimulating Hormone versus Thyroid Hormone Withdrawal for Radioactive Iodine Treatment of Differentiated Thyroid Cancer with Nodal Metastatic Disease

**DOI:** 10.1155/2016/6496750

**Published:** 2016-02-09

**Authors:** Robert M. Wolfson, Irina Rachinsky, Deric Morrison, Al Driedger, Tamara Spaic, Stan H. M. Van Uum

**Affiliations:** ^1^Department of Diagnostic Imaging, Schulich School of Medicine and Dentistry, Western University, London, ON, Canada N6A 5W9; ^2^Department of Medicine, Schulich School of Medicine and Dentistry, Western University, London, ON, Canada N6A 4V2

## Abstract

*Introduction.* Recombinant human thyroid stimulating hormone (rhTSH) is approved for preparation of thyroid remnant ablation with radioactive iodine (RAI) in low risk patients with well differentiated thyroid cancer (DTC). We studied the safety and efficacy of rhTSH preparation for RAI treatment of thyroid cancer patients with nodal metastatic disease.* Methods.* A retrospective analysis was performed on 108 patients with histopathologically confirmed nodal metastatic DTC, treated with initial RAI between January 1, 2000, and December 31, 2007. Within this selected group, 31 and 42 patients were prepared for initial and all subsequent RAI treatments by either thyroid hormone withdrawal (THW) or rhTSH protocols and were followed up for at least 3 years.* Results.* The response to initial treatment, classified as excellent, acceptable, or incomplete, was not different between the rhTSH group (57%, 21%, and 21%, resp.) and the THW group (39%, 13%, and 48%, resp.; *P* = 0.052). There was no significant difference in the final clinical outcome between the groups. The rhTSH group received significantly fewer additional doses of RAI than the THW group (*P* = 0.03).* Conclusion.* In patients with nodal-positive DTC, preparation for RAI with rhTSH is a safe and efficacious alternative to THW protocol.

## 1. Introduction

Following initial surgery, the recurrence risk for differentiated thyroid cancer (DTC) is defined as low, intermediate, or high [1]. Treatment with radioactive iodine (RAI) is also used for remnant ablation and for patients with local and distant metastases [1]. Administration of RAI requires increased TSH to maximize RAI uptake in benign or malignant thyroid tissue. Traditionally, this has been achieved by thyroid hormone withdrawal (THW). Several studies, including one prospective study [2], demonstrated the effectiveness of recombinant human thyroid stimulating hormone (rhTSH) for thyroid remnant ablation. In 2007 this resulted in approval by the FDA for preparation of thyroid remnant ablation with radioactive iodine (RAI) in low risk patients with DTC and no evidence of metastatic disease. In Canada, rhTSH was approved for this indication in 2009. In contrast, rhTSH has not been approved in the US or Canada for use in RAI treatment of recurrent disease or distant metastases [3]. Thus, THW, rather than rhTSH, is recommended for patients with intermediate or high recurrence risk. The use of rhTSH for these groups is considered off-label.

The conventional preparation method with THW causes hypothyroidism which is symptomatic in almost all patients. Luster et al. [4] reported that 92% of patients developed hypothyroid symptoms, and almost half of them sought medical attention because of this. These transient effects have not been described in relation to rhTSH administration, and it has no known long-term side effects. Recently Hugo et al. [5] reported that, compared to THW, rhTSH preparation for RAI treatment resulted in similar final clinical outcomes across a wide range of risks of recurrence and risks of thyroid cancer related death. In our centre, as early as 2000, we started offering rhTSH to patients who potentially could have higher incidence of hypothyroid complications, for example, patients with known depression and previous suicide attempts, professional drivers, and patients with heart or renal failure. Thus, we also prepared some patients with cervical lymph node metastases with rhTSH rather than with THW for RAI treatment. In the present retrospective study, we compare the effect of THW with that of rhTSH for preparation for RAI treatment in newly diagnosed thyroid cancer patients who had intermediate recurrence risk based on histological presence of cervical lymph node metastases at presentation. We analysed both initial response to treatment and clinical status after long-term follow-up.

## 2. Methods

Patients were recruited from the thyroid cancer clinics at the London Health Sciences Centre, the major referral centre for patients residing in Southwestern Ontario, Canada. Since 1998, all thyroid cancer patients attending our thyroid cancer clinics have been invited to participate in a thyroid cancer registry. More than 95% of invited patients provided written informed consent for inclusion in this registry. The study was approved by the Health Sciences Research Ethics Board of the University of Western Ontario.

For the present study, we searched the registry for all newly diagnosed thyroid cancer patients who were seen at our institution between January 1, 2000, and December 31, 2007. Patients were included if they had well DTC with nodal metastatic disease diagnosed on histopathology at the time of their thyroidectomy. They needed to have received RAI treatment at least once. In addition, we required that any subsequent RAI treatments were administered using the same protocol (THW or rhTSH administration) that was used for the initial therapy. The choice to use rhTSH was made jointly by the patient and the treating physician. For the treatment described in this study, the use of rhTSH was off-label. All patients were followed up for a minimum of 3 years or until death. Clinical follow-up occurred every 6–12 months for all patients and included baseline (suppressed) thyroglobulin (Tg), Tg antibody levels, and annual neck ultrasound. Stimulated thyroglobulin Tg test was performed within 2 years of initial RAI treatment.

For evaluation of the response to initial treatment and the clinical status at the time of final follow-up, we applied the criteria defined by Hugo et al. [5]. Briefly, the response to initial treatment was classified as* excellent* when both suppressed and stimulated Tg were <1 ng/mL, and there was no evidence of disease on neck ultrasound, whole body iodine scan, or CT scan.* Acceptable* response was defined as suppressed Tg <1 ng/mL, stimulated Tg 1–10 ng/mL, and/or equivocal findings on diagnostic imaging.* Incomplete* response was defined as suppressed Tg >1 ng/mL, stimulated Tg >10 ng/mL, and/or evidence of persistent disease on diagnostic imaging. For final outcome, patients were classified according to their status at last follow-up.* No evidence of disease* was defined as suppressed Tg <1 ng/mL, no detectable anti-Tg antibody, and no structural evidence of disease on clinical examination or radiological studies.* Persistent disease* was defined as suppressed Tg values >1 ng/mL, stimulated Tg values >2 ng/mL, and/or evidence of persistent disease in structural or functional imaging.

*Recurrent disease* was defined as suppressed Tg >1 ng/mL and/or structural or functional evidence of disease identified following a period of no evidence of disease.

Patients who died from thyroid cancer were categorized as* death due to thyroid cancer.*

Results are described as mean ± SD or % as appropriate. The Student's *t*-test was used for group comparison of quantitative data. Categorical comparison of the groups was done using chi square testing and Fisher's exact test as appropriate (SPSS version 20.0). A *P* value <0.05 was considered statistically significant.

## 3. Results

We identified 108 patients with newly diagnosed well DTC with nodal metastatic disease diagnosed on histopathology at the time of thyroidectomy. All patients had received at least one RAI treatment, 56 patients were initially prepared with rhTSH, and 52 patients were prepared with THW for the first RAI treatment. Of the 56 patients in the rhTSH group, 7 were excluded because they underwent THW at one or more subsequent treatments, and 7 patients had insufficient follow-up. Of the 52 patients in the THW group, 8 were excluded because they received rhTSH at one or more subsequent treatments, and 13 patients had insufficient follow-up. Therefore we included 42 patients in the rhTSH group and 31 patients in the THW group. Clinical follow-up occurred every 6–12 months and laboratory and imaging studies were done as described above. In addition, at least one neck ultrasound and one stimulated Tg test were performed within the first 2 years following initial RAI. Patients were followed up clinically until they were deemed disease-free (undetectable or negative Tg, U/S) and discharged to a local endocrinologist or had succumbed to death.

Baseline parameters of both THW and rhTSH groups are presented in Table 1(a). Patients in the rhTSH group were older than those in the THW group (*P* = 0.039). However, there was no difference in the proportion of patients younger than 45 years between the two groups (*P* = 0.075). There were less male patients (19%) in the THW group than in the rhTSH group (40%), although this difference did not reach statistical significance (*P* = 0.08). There was no difference in histological subtype or thyroid cancer size at presentation between groups.

Information on staging and recurrence risk is presented in Table 1(b). There was no statistically significant difference in TNM staging, AJCC staging, or ATA risk between THW and rhTSH groups. The duration of follow-up was longer in the THW group than in the rhTSH group (*P* = 0.01), but there was no difference in total RAI activity that had been administered.

**Figure 1:**
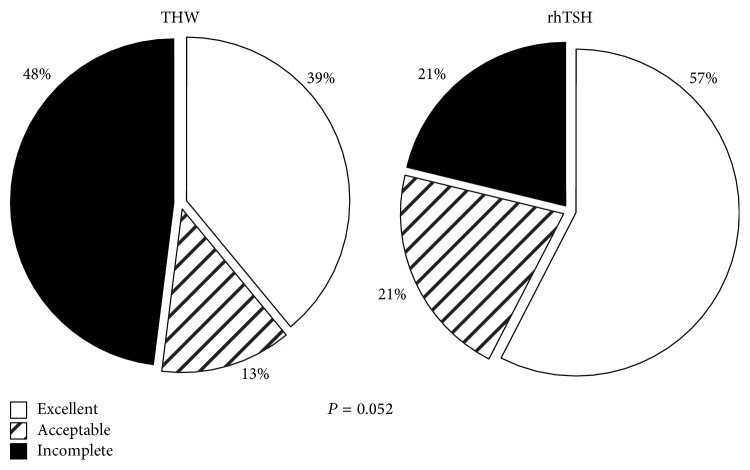
Response to initial treatment with radioactive iodine, specified according to preparation with thyroid hormone withdrawal (THW) versus preparation with recombinant human TSH (rhTSH).

**Figure 2:**
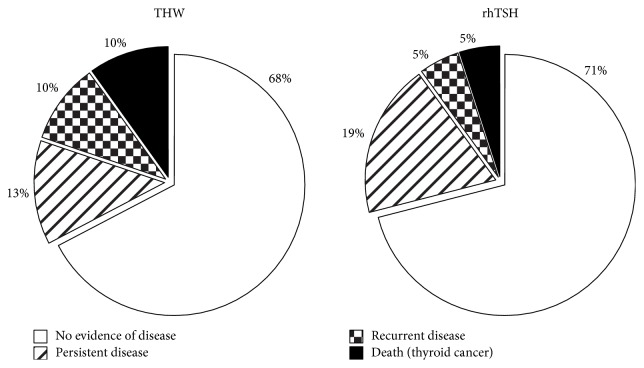
Final clinical outcome specified according to preparation with thyroid hormone withdrawal (THW) versus preparation with recombinant human TSH (rhTSH).

The response to initial treatment showed a trend towards superiority in the rhTSH group compared to the THW group (*P* = 0.052). The majority of patients in the rhTSH group had an excellent response, while almost half of the patients in the THW group had an incomplete response (Figure 1).  Table 2 presents further information on initial and additional treatments and clinical outcome at follow-up. Patients in the THW group received more additional RAI treatments than the rhTSH group. There was no difference between groups with respect to the outcome as assessed at the last visit (Figure 2). In both groups about 70% had no evidence of persistent disease, while 3 patients died of thyroid cancer in the THW group and 2 in the rhTSH group (Table 2).

The need for additional RAI treatments was higher in patients with N1b stage (41%) than in patients with N1a stage (31%; *P* < 0.05).

## 4. Discussion

In this study we compared THW with rhTSH for preparation of RAI treatment in DTC patients with intermediate recurrence risk and did not find any difference in clinical outcome after about seven years of follow-up between rhTSH and THW preparation. Over the last years several groups have compared rhTSH preparation with THW for initial RAI. Sabra et al. [3] reviewed 9 studies that compared these two preparation modalities with respect to the effect of first adjuvant RAI treatment on the percentage of patients that achieved an undetectable stimulated thyroglobulin (<2 ng/mL). They found no difference between rhTSH and THW preparation; after 1-2 years, nearly 75% of patients were classified as having no evidence of disease. However, the stimulated thyroglobulin test is an intermediate endpoint and the follow-up duration was relatively short.

Focusing on clinical outcomes over longer follow-up time, Rosario et al. [6] reported clinical outcome after 5-year follow-up in a cohort of 276 patients with N1 disease and found no difference between rhTSH and THW prepared group.

For patients with ATA intermediate risk, Hugo et al. [5] also found no difference in final clinical outcome between 291 patients prepared with withdrawal and 141 patients prepared with rhTSH. A more recent study by Molinaro et al. [7] prospectively evaluated 10-year follow-up in DTC patients. While the number of patients with ATA intermediate recurrence risk was relatively small, 11 in the rhTSH group and 9 in the THW group, they found no difference in final clinical outcome between preparation modalities. Similar results have also been reported for patients with RAI avid-metastatic disease by Klubo-Gwiezdzinska et al. [8], who found that rhTSH preparation and THW withdrawal resulted in similar clinical outcomes of RAI treatment.

In our patients the response to initial therapy showed a trend towards being more favorable in the rhTSH group than in the THW group. In addition, the rhTSH group required significantly fewer additional RAI treatments than the THW group, despite the increased prevalence of 2 risk factors for more aggressive disease (higher age and higher percentage of male patients) in the rhTSH group. Similar results have been found by other investigators. For patients with an intermediate ATA recurrence risk, Hugo et al. [5] reported an excellent response in 43.1 and 30.8%, an acceptable response for 26.6 and 24.8%, and an incomplete response for 30.3 and 44.4% for rhTSH and THW groups, respectively. They also found that patients in the THW group were more likely to require additional RAI treatments than patients treated with rhTSH. For patients with an intermediate ATA recurrence risk, Sabra et al. [3] reported an excellent response in 43% of rhTSH patients and 31% of THW patients (*P* = 0.03).

The 2015 ATA guidelines state in recommendation 34 that, for patients with ATA low and intermediate DTC, rhTSH stimulation is an acceptable alternative to THW for initial RAI treatment/remnant ablation [9]. The results of the present study support this. With respect to further RAI treatments, the 2015 ATA guideline suggests there is insufficient evidence to support use of rhTSH for RAI treatment. Our study suggests that in this situation use of rhTSH results in equal outcomes as compared to THW.

We are not sure why there was a trend towards higher incidence of male patients in the rhTSH group than in the THW group in our study. The age bias is most likely due to a higher prevalence of medical comorbidities in older patients, making the risks of hypothyroidism with the THW protocol higher, resulting in choosing rhTSH for more of these patients.

As these studies in patients with ATA intermediate recurrence risk suggest that there is no difference in longer-term clinical outcome between THW and rhTSH preparation and there is either no difference or improved response to initial treatment, it becomes important to compare side effects of the two preparation methods. THW is associated with hypothyroidism and a decrease in QOL. Luster et al. [4] reported that 92% of patients had symptomatic and 85% had multisymptomatic hypothyroidism. In 2006, Schroeder et al. [10] reported that THW is associated with significant decline in quality of life as assessed by SF-36 questionnaire, an effect that was not found after rhTSH administration. An important study was reported by Nygaard et al. [11] who performed a double blind, placebo-controlled randomized cross-over study comparing liothyronine L-T3 THW for 10 days with rhTSH in 56 patients with DTC receiving RAI treatment. Their primary outcome was QOL as assessed by SF-36 questionnaire, and THW was associated with a significantly worse QOL in 2 domains, social functioning and mental health. Indeed, severe hypothyroidism is associated with reversible depression, slowed reaction time, and decreased fine-motor performance and processing speed [12]. Thus, during severe hypothyroidism, patients should be cautioned against activities that could be affected by the effects of hypothyroidism, such as driving motor vehicles.

There are several limitations pertaining to the present study, including its retrospective, nonrandomized, nonblinded design. In addition, the number of patients was relatively small. Recent studies suggest that the outcome and treatment choice may be affected by the extent of node positivity (N1a versus N1b) and probably the lymph node size [9]. We also found that additional RAI treatment was required in more patients with N1b status than patients with N1a status. Regarding the size of lymph nodes, this was reported inconsistently during the study period, so that we are not able to perform this analysis.

Strengths of this study relate to its inclusion of patients from a well-defined regional area, reducing referral bias and the inclusion of a homogeneous group of patients all with pathologically proven cervical lymph node metastasis. In addition, all patients received only one method of preparation, either rhTSH or THW, for the initial and all subsequent RAI treatments. Finally, all patients were followed up on the long term in similar fashion in one single centre.

The 2015 ATA guideline definitions for assessment of clinical status during follow-up [9] vary slightly from the definitions in our study. The ATA guideline definition of excellent response is the same as that in our study. The ATA guideline differentiates between biochemical and structural incomplete response, while in our study these have been grouped together. The “indeterminate response” is defined in the 2015 ATA guideline as “non-specific, biochemical or structural findings which cannot be confidently classified as either benign or malignant” and is essentially the same as the “acceptable response” in our study. Based on the lower limit of detectable thyroglobulin in the assay used at the time of our study, we determined study-specific biochemical criteria to define clinical status, for example, “no evidence of disease” or “persistent” disease. In addition, we developed a specific definition for recurrent disease, which was important for the primary objective of our study.

The optimal management of regional lymph node metastases and ATA intermediate risk patients in thyroid cancer remains to be determined [13]. A recent study suggests that the presence of cervical lymph node metastasis increases the risk for recurrence of thyroid cancer and mortality [14], and another retrospective study suggests that treating these patients with RAI decreases mortality [15]. This indicates that it remains extremely important to determine how adjuvant RAI can be most effective with least side effects. The present study lends further support to the notion that use of rhTSH in thyroid cancer patients with nodal metastatic disease is associated with similar long-term outcomes while perhaps improving initial response to treatment. This suggests that it is time for a well-designed prospective randomized study comparing the effect of rhTSH and THW on the initial response, clinical outcomes, and short- and long-term side effects in patients with intermediate recurrence risk.

## Figures and Tables

**(a):** 

Parameter	THW (*n* = 31)	rhTSH (*n* = 42)	*P* value
Age at ablation			
Mean ± SD (years)	38.2 ± 12.4	45.7 ± 16.2	0.039
<45	23 (74%)	22 (52%)	0.075
>45	8 (26%)	20 (48%)	

Male	6 (19%)	17 (40%)	
Female	25 (81%)	25 (60%)	0.08

Histology			0.92
Papillary thyroid cancer	29 (94%)	41 (98%)	
Classical variant	9 (29%)	10 (24%)	
Follicular variant	6 (19%)	9 (21%)	
Mixed classical/follicular variant	4 (13%)	6 (14%)	
Aggressive variant	3 (10%)	5 (12%)	
Cystic variant	1 (3%)	0 (0%)	
Variant not available	6 (19%)	11 (26%)	
Follicular thyroid cancer	0 (0%)	0 (0%)	
Hurthle cell thyroid cancer	0 (0%)	0 (0%)	
Poorly differentiated cancer	1 (3%)	1 (3%)	
Thyroid cancer not specified	1 (3%)	0 (0%)	

Size of primary thyroid cancer (cm)	2.9 ± 1.5	2.2 ± 1.5	0.09
Median	2.6	2.0	

**(b):** 

Parameter	THW (*n* = 31)	rhTSH (*n* = 42)	*P* value
Tumor staging			0.59
1	7 (23%)	13 (31%)	
2	6 (19%)	4 (10%)	
3	15 (48%)	22 (52%)	
4a	3 (10%)	3 (7%)	
4b	0 (0%)	0 (0%)	

Nodal staging			0.73
1a	21 (68%)	30 (71%)	
1b	10 (32%)	12 (29%)	

Metastatic staging			0.24
0	30 (97%)	42 (100%)	
1	1 (3%)	0 (0%)	

AJCC staging			0.17
1	22 (71%)	22 (52%)	
2	1 (3%)	0 (0%)	
3	5 (16%)	13 (31%)	
4a	3 (10%)	7 (17%)	
4b	0 (0%)	0 (0%)	
4c	0 (0%)	0 (0%)	

ATA recurrence risk			0.33
Low	0 (0%)	0 (0%)	
Intermediate	23 (74%)	37 (88%)	
High	8 (26%)	5 (12%)	

Administered RAI activity (GBq)	4.58 ± 1.49	4.52 ± 0.92	0.48

Duration of follow-up (years)	8.6 ± 2.4	6.8 ± 2.1	0.01

**Table 2:** Response to initial treatment, additional treatments, and outcome.

Parameter	THW (*n* = 31)	rhTSH (*n* = 42)	*P* value
Response to initial treatment			0.052
Excellent	12 (39%)	24 (57%)	
Acceptable	4 (13%)	9 (21%)	
Incomplete	15 (48%)	9 (21%)	

Additional surgeries^*∗*^			0.44
None	21 (68%)	33 (79%)	
1	6 (19%)	7 (17%)	
2	4 (13%)	2 (5%)	

Additional RAI treatments^#^			0.03
None	17 (55%)	28 (67%)	
1	9 (29%)	14 (33%)	
≥2	5 (16%)	0 (0%)	

Distant metastasis at any time			0.21
0	27 (87%)	40 (95%)	
1	4 (13%)	2 (5%)	

Clinical status at last visit			0.62
No evidence of disease	21 (68%)	30 (71%)	
Persistent disease	4 (13%)	8 (19%)	
Recurrent disease	3 (10%)	2 (5%)	
Death due to thyroid cancer	3 (10%)	2 (5%)	

^*∗*^Additional surgery usually consisted of removal of one or more lymph nodes.

^#^The dose for each additional treatment, a fixed dose of 5.5 GBq (150 mCi) of 131-I.
